# The bZIP Transcription Factor ZIP-11 Is Required for the Innate Immune Regulation in *Caenorhabditis elegans*

**DOI:** 10.3389/fimmu.2021.744454

**Published:** 2021-11-05

**Authors:** Zhongfan Zheng, Yilixiati Aihemaiti, Junqiang Liu, Muhammad Irfan Afridi, Shengmei Yang, Xiumei Zhang, Yongfu Xu, Chunhong Chen, Haijun Tu

**Affiliations:** ^1^ State Key Laboratory of Chemo/Biosensing and Chemometrics, College of Biology, Hunan University, Changsha, China; ^2^ Shenzhen Research Institute, Hunan University, Shenzhen, China

**Keywords:** innate immunity, bZIP transcription factor, intestine, ATF4/ZIP-11, PMK-1/p38, CEBP-2

## Abstract

Innate immunity is the first line of host defense against pathogen infection in metazoans. However, the molecular mechanisms of the complex immune regulatory network are not fully understood. Based on a transcriptome profiling of the nematode *Caenorhabditis elegans*, we found that a bZIP transcription factor ZIP-11 was up-regulated upon *Pseudomonas aeruginosa* PA14 infection. The tissue specific RNAi knock-down and rescue data revealed that ZIP-11 acts in intestine to promote host resistance against *P. aeruginosa* PA14 infection. We further showed that intestinal ZIP-11 regulates innate immune response through constituting a feedback loop with the conserved PMK-1/p38 mitogen-activated protein signaling pathway. Intriguingly, ZIP-11 interacts with a CCAAT/enhancer-binding protein, CEBP-2, to mediate the transcriptional response to *P. aeruginosa* PA14 infection independently of PMK-1/p38 pathway. In addition, human homolog ATF4 can functionally substitute for ZIP-11 in innate immune regulation of *C. elegans*. Our findings indicate that the ZIP-11/ATF4 genetic program activates local innate immune response through conserved PMK-1/p38 and CEBP-2/C/EBPγ immune signals in *C. elegans*, raising the possibility that a similar process may occur in other organisms.

## Introduction

The innate immune system is an evolutionarily conserved defense mechanism against microbial infection, which elicits immediate defense and generates long-term adaptive immunity (1). Innate immunity can be divided into two distinct but overlapping components: constitutive and inducible immunity. Constitutive immunity includes basally expressed immune effectors that allow host to response to the invading pathogens instantaneously (2, 3). The inducible immune response includes three stages in general (4). First, the invading pathogen is recognized by the host pattern recognition receptors (PRRs). Next, the infection signals are processed and delivered *via* the activation or suppression of concerned signaling pathways. Finally, the up or down-regulated immune effectors accelerate to eliminate the pathogen or alleviate its effect on the host.

The nematode *Caenorhabditis elegans* is a tractable genetic model to study innate immunity (5). Based on forward and reverse genetic analyses, several evolutionarily conserved signaling pathways required for *C. elegans* response to diverse pathogens have been identified, including a p38 mitogen-activated protein kinase (MAPK) pathway, an insulin-like receptor (IGF-1) pathway, a transforming growth factor (TGF-β) pathway and a programmed cell death (PCD) pathway (6–9). Of central importance is the PMK-1/p38 pathway. Previous studies showed that PMK-1 is regulated by a phosphorylation cascade controlled by a Toll/IL-1 receptor domain protein, TIR-1, leading to the activation of a MAPKKK called NSY-1, which further activates a MAPKK called SEK-1, culminating in the phosphorylation of PMK-1 (6, 10). The homolog of *C. elegans* NSY-1-SEK-1-PMK-1 MAPK signaling pathway is ASK-1-MKK3/6-p38 MAPK in mammals. Recent findings have revealed that multiple upstream signaling such as the human RIO kinase homolog RIOK-1, the neural GPCR OCTR-1, dopaminergic signaling, and proline catabolism modulate innate immune response against *P. aeruginosa* PA14 infection through the PMK-1/p38 pathway in cell-autonomous or cell-non-autonomous manner (11–14).

The basic region leucine zippers (bZIPs) are evolutionarily conserved transcription factors in eukaryotic organisms (15). bZIPs were identified from eukaryotic genomes by a basic DNA binding region followed by a leucine-zipper coiled-coil motif, *via* which the bZIP proteins can form homodimers and heterodimers, further influences the DNA sites that can be bound (16). The bZIP proteins are involved in diverse biological processes such as development, reproduction, cell cycle, stress response, and immune regulation (17, 18). Up to date, about 33 bZIP transcription factors have been identified in *C. elegans* (19), which are predominantly expressed in intestinal tissue and widely participate in the regulatory network of innate immunity. Among these immune-related bZIP transcription factors, some are directly activated or suppressed by the upstream kinases or phosphatase, such as SKN-1, CEBP-1 and ATF-7 (20–22), while some members were involved in innate immune regulation through the changes in expression levels, such as ZIP-2 and ATFS-1 (23, 24).

In this study, we identified an evolutionarily conserved bZIP transcription factor ZIP-11/ATF4 as a novel innate immune modulator. Intestinal ZIP-11 promotes host resistance against pathogens through constituting a feedback loop with PMK-1/p38 pathway. In parallel, ZIP-11 acts with a CCAAT/enhancer-binding protein, CEBP-2, to enhance resistance pathogen infection independently of PMK-1/p38 pathway. These ZIP-11-involved innate immune response pathways promote infection tolerance upon *P. aeruginosa* PA14 infection, which might be conserved across species and expands our knowledge of infectious disease tolerance.

## Results

### *P. aeruginosa* PA14 Infection Induces the Expression of ZIP-11

**Figure 1:**
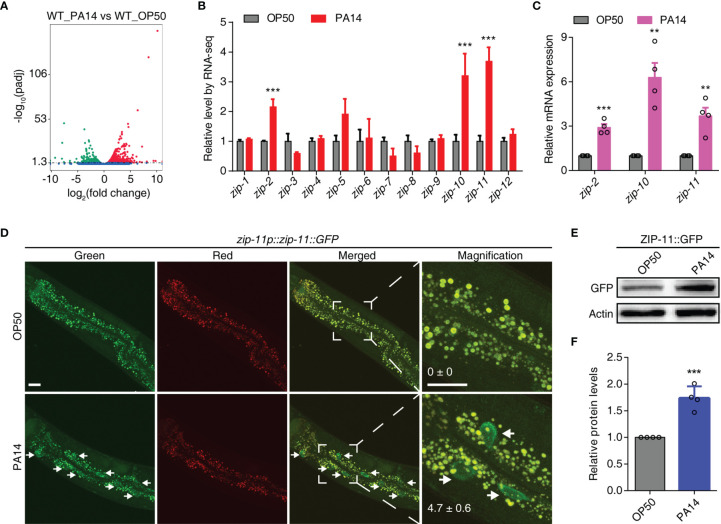
*P. aeruginosa* PA14 infection induces the expression of ZIP-11. **(A)** Volcano plot of RNA-seq showing different regulated genes (up-regulated genes in red; down-regulated genes in green; no significantly-changed genes in blue) of wild type worms upon *P. aeruginosa* PA14 infection. **(B)** Quantification of RNA-seq reads indicating up-regulation of *zip-2*, *zip-10* and *zip-11* but not the other members of the *zip* class genes. **(C)** qRT-PCR analysis of *zip-2*, *zip-10* and *zip-11* gene expression in WT worms exposed to *E. coli* OP50 or *P. aeruginosa* PA14. **(D)** Representative images of the *zip-11p::zip-11::GFP* worms exposed to *E. coli* OP50 or *P. aeruginosa* PA14. Right panels are higher magnification. Mean percentages of ZIP-11::GFP nuclear accumulation in the anterior portion of intestinal tissue (white arrows) are indicated. n ≥ 14. Scale bar: 20 μm. **(E)** Western blots of the *zip-11p::zip-11::GFP* worms exposed to *E. coli* OP50 or *P. aeruginosa* PA14 by using anti-GFP antibody. **(F)** Quantification of the protein expression levels using the software program ImageJ. Data are presented as mean ± SEM. Statistical significance was determined by Student’s *t*-test **(C, F)**. **p < 0.01, ***p < 0.001 compared with respective controls unless specifically indicated.

To better understand the mechanisms of the host responses to pathogenic infection, we performed transcriptome RNA-sequencing (RNA-seq) of wild-type (N2) *C. elegans* fed on *E. coli* OP50 or exposed to *P. aeruginosa* PA14. Differential expression analyses of triplicate samples demonstrated that 643 genes were up-regulated and 582 genes were down-regulated upon *P. aeruginosa* PA14 exposure as compared to the gene expression levels in *C. elegans* fed on *E. coli* OP50 (**Figure 1A**; **Tables S1**, **S2 in Supplementary Material**). Subsequently, we compared our RNA-seq data with two previous studies (25, 26). The results show that about half of the differentially expressed genes in either of the previous studies were commonly regulated by *P. aeruginosa* infection as compared to that in this study (**Figure S1A in Supplementary Material**), which give us a notion that despite the known immune molecules and pathways, further efforts are needed to explore the regulatory network involved in innate immunity. Considering the mounting evidence shows that bZIP transcription factors play crucial roles in innate immune regulation, here, we focused on the immune function of *zip* class genes. Out of 12 members of the *zip* family in *C. elegans*, *zip-2*, *zip-10* and *zip-11* were up-regulated upon *P. aeruginosa* PA14 infection (**Figure 1B**; **Table S3 in Supplementary Material**) and those results were verified by quantitative real-time PCR (qRT-PCR) (**Figure 1C**). ZIP-2 has been identified as a key mediator of infection-induced gene expression (27). Translational block caused by PA14-delivered ToxA can trigger an increase in protein level of ZIP-2 (23). Meanwhile, ZIP-10 acts downstream of the TGF-β signal pathway ligand DBL-1 and the SMAD transcription factor SMA-9 to regulate the body size and the male tail morphogenesis of *C. elegans* (28). Moreover, the exposure to pathogenic bacteria induces ZIP-10 expression (25), indicating ZIP-10 may also play an important role in innate immunity.

Since ZIP-11 has not been characterized yet, we focused on this bZIP transcription factor to investigate if and how it mediates the innate immunity of *C. elegans*. Using a tagged *zip-11p::zip-11::GFP::FLAG* allele in an integrated transgenic strain, we observed that the number of intestinal nuclei with visible ZIP-11::EGFP was significantly increased in *P. aeruginosa* PA14 exposed worms than that in *E. coli* OP50 fed worms (**Figure 1D**). Likely because of the low baseline expression level of ZIP-11, the EGFP fluorescence signal of the normal maintained *C. elegans* was barely visible in intestinal tissue. Moreover, the western blot results also showed that *P. aeruginosa* PA14 infection dramatically upregulated the expression level of ZIP-11 protein (**Figures 1E, F**). These results demonstrate that pathogen infection stimulates ZIP-11 expression which assist our presumption of possible role of ZIP-11 in innate immune response.

### ZIP-11 Functions in Intestine to Promote Innate Immunity Against *P. aeruginosa* PA14 Infection

**Figure 2:**
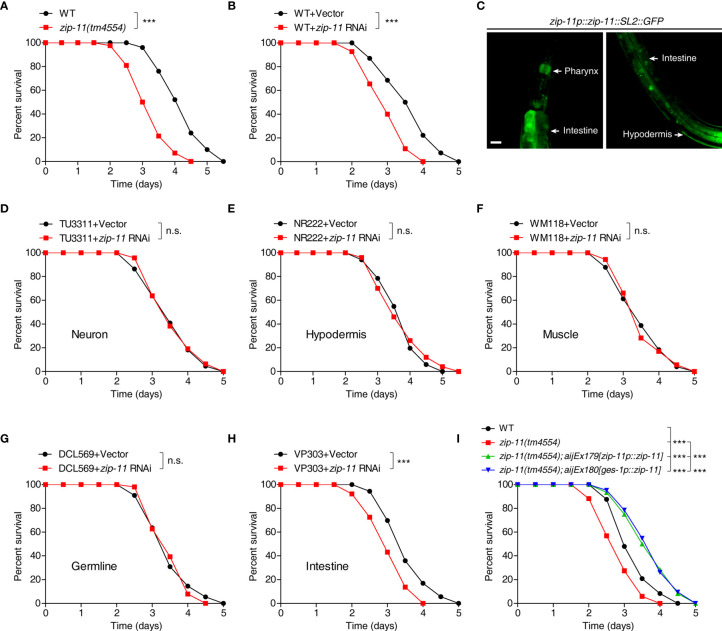
Intestinal ZIP-11 activates innate immunity against *P. aeruginosa* PA14 infection. **(A)** WT worms and *zip-11(tm4554)* worms were exposed to *P. aeruginosa* PA14 and scored for survival. **(B)** WT worms fed with vector control or *zip-11* RNAi bacteria were exposed to *P. aeruginosa* PA14 and scored for survival. **(C)** High magnification images of *zip-11p::zip-11::SL2::GFP* transgenic worms show *zip-11* is expressed in intestine, pharynx, and hypodermis. Scale bar: 20 μm. **(D–H)** Tissue-specific RNAi worm strains including TU3311/neuron **(D)**, NR222/hypodermis **(E)**, WM118/muscle **(F)**, DCL569/germline **(G)** and VP303/intestine **(H)** fed with vector control or *zip-11* RNAi bacteria were exposed to *P. aeruginosa* PA14 and scored for survival. **(I)** WT, *zip-11(tm4554)*, *zip-11(tm4554);aijEx179[zip-11p::zip-11]* and *zip-11(tm4554);aijEx180[ges-1p::zip-11]* worms were exposed to *P. aeruginosa* PA14 and scored for survival. Statistical significance was determined by log-rank test for killing assays. Please see **Table S4 in Supplementary Material** for detailed statistical analysis of killing assay data. ***p < 0.001; n.s., not significant.

To test if ZIP-11 acts in innate immune response, we first examined the survival of ZIP-11 deficient worms upon *P. aeruginosa* PA14 infection. We found that *zip-11(tm4554)* null mutant worms exhibited reduced resistance to killing by *P. aeruginosa* PA14 as compared to wild-type (**Figure 2A**). Furthermore, the sensitive phenotype to *P. aeruginosa* PA14 infection was also observed in worms treated with *zip-11* RNA interference (RNAi) (**Figure 2B**). These data suggest that the loss function or the suppression of ZIP-11 signaling impairs the innate immunity of *C. elegans*. Certain mutants susceptible to pathogen infection exhibit sensitivity to pathogen accumulation, while others exhibit reduced endurance to pathogen (11). Here, the numbers of bacterial cells in the intestine of *zip-11* mutants were comparable to those in WT worms (**Figure S1B in Supplementary Material**). Moreover, compared to WT worms, *zip-11(tm4554)* worms exhibited a similar accumulation pattern of *P. aeruginosa*/green fluorescent protein (GFP) (**Figure S1C in Supplementary Material**). To ask whether the feeding or defecation behavior in *zip-11(tm4554)* animals may account for the immune phenotype, the pumping rate and enteric muscle contraction (EMC) cycle in the intestine were detected. We observed that there was no difference in these behaviors between *zip-11(tm4554)* and WT worms (**Figures S1D, S1E in Supplementary Material**). In addition, *zip-11(tm4554)* worms showed the equivalent brood size as that of WT worms (**Figure S1F in Supplementary Material**), suggesting that the observed sensitivity to *P. aeruginosa* infection in *zip-11(tm4554)* worms is not caused by the confounding effects varying on worm fecundity. There was no difference in survival rate between *zip-11(tm4554)* and WT worms that were fed with heat-killed *P. aeruginosa* (**Figure S1G in Supplementary Material**). Thus, ZIP-11 activates immune response to promote the endurance to living pathogen bacteria without altering the basic health of *C. elegans*.

Although ZIP-11 expression was only visible in the intestinal nuclei of worms exposed to *P. aeruginosa* using the translational reporter *zip-11p::zip-11::GFP*, we generated the *zip-11* transcriptional reporter *zip-11p::zip-11::SL2::GFP* to monitor the expression pattern of *zip-11* in *C. elegans*. Intriguingly, the GFP signal was detectable not only in intestine but also in pharynx and hypodermis tissue (**Figure 2C**; **Figure S2 in Supplementary Material**), indicating that ZIP-11 may play a regulatory role in multiple biological processes. In order to determine the site of action of ZIP-11 and clarify the tissue specificity of ZIP-11 in terms of immune regulation to *P. aeruginosa* infection, the killing assay of worms treated with *zip-11* RNAi restricted to specific tissue was performed. The specificity of tissue-specific RNAi worm strains was confirmed by tissue-specific RNAi clones, which acted only in the corresponding tissues and demonstrated specific phenotypes (29). With tissue-specific RNAi worms, only the knockdown of *zip-11* in intestine reduced the resistance to *P. aeruginosa* infection (**Figure 2H**), of which the phenotype was in accordance with that observed in *zip-11* loss-function and whole-body knockdown worms. However, suppressing *zip-11* expression in the neuron (**Figure 2D**), or hypodermis (**Figure 2E**), or muscle (**Figure 2F**), or germline (**Figure 2G**) did not alter the survival rate of worms upon *P. aeruginosa* infection. Additionally, we found that overexpression of ZIP-11 under the control of endogenous *zip-11* or intestinal specific *ges-1* promoter in *zip-11* mutant background converted the sensitive phenotype to the resistant state (**Figure 2I**). Taken together, these results support a role of ZIP-11 in activating a protective transcriptional response in the intestinal tissue of *C. elegans* to *P. aeruginosa* infection.

### ZIP-11 Activates Innate Immunity *via* PMK-1/p38 MAPK Pathway

**Figure 3:**
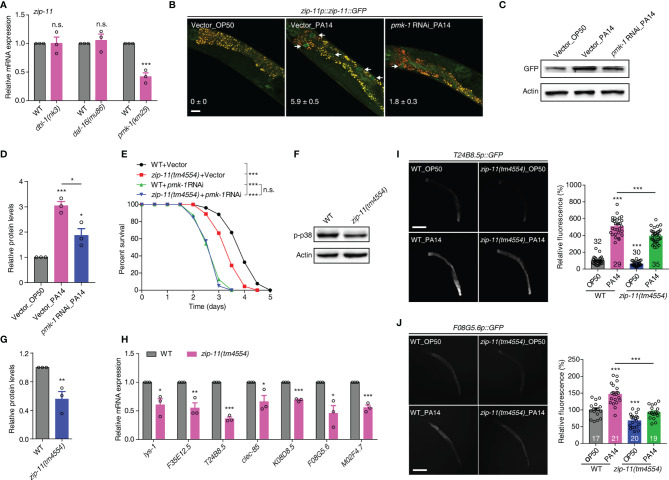
The conserved PMK-1/p38 pathway is required for the attenuated resistance to *P. aeruginosa* infection of *zip-11* mutants. **(A)** qRT-PCR analysis of *zip-11* expression in WT, *dbl-1(nk3)*, *daf-16(mu86)* or *pmk-1(km25)* worms. **(B)** Representative images of the *zip-11p::zip-11::GFP* worms exposed to *E. coli* OP50 or *P. aeruginosa* PA14 after being fed with vector control or *pmk-1* RNAi bacteria. Mean percentages of ZIP-11::GFP nuclear accumulation in the anterior portion of intestinal tissue (white arrows) are indicated. n ≥ 15. Scale bar: 20 μm. **(C)** Western blots of *zip-11p::zip-11::GFP* worms exposed to *E. coli* OP50 or *P. aeruginosa* PA14 after being fed with vector control or *pmk-1* RNAi bacteria. **(D)** Quantification of the protein expression levels using the software program ImageJ. **(E)** WT worms and *zip-11(tm4554)* worms fed with vector control, *pmk-1* RNAi bacteria were exposed to *P. aeruginosa* PA14 and scored for survival. **(F)** Western blot analysis of p38 phosphorylation levels in WT and *zip-11(tm4554)* worms. **(G)** Quantification of the protein expression levels using the software program ImageJ. **(H)** qRT-PCR analysis of the expression of PMK-1-dependent genes in WT and *zip-11(tm4554)* worms. **(I)** Representative images and quantification of *T24B8.5p::GFP* in WT and *zip-11(tm4554)* mutant background exposed to *E. coli* OP50 or *P. aeruginosa* PA14. The number of animals analysed is indicated. Scale bar: 200 μm. **(J)** Representative images and quantification of *F08G5.6p::GFP* in WT and *zip-11(tm4554)* mutant background exposed to *E. coli* OP50 or *P. aeruginosa* PA14. The number of animals analysed is indicated. Scale bar: 200 μm. Data are presented as mean ± SEM. Statistical significance was determined by log-rank test **(E)** or Student’s *t*-test **(A, D, G-J)**. *p < 0.05, **p < 0.01, ***p < 0.001 compared with respective controls unless specifically indicated; n.s., not significant. Please see **Table S4 in Supplementary Material** for detailed statistical analysis of killing assay data.

Upon *P. aeruginosa* infection, *C. elegans* mounts protective responses by triggering evolutionarily conserved innate immune pathways, such as the DBL-1/TGF-β, DAF-16/FOXO and PMK-1/p38 MAPK pathways (6–8). To gain insights into the molecular mechanism of ZIP-11 regulating *C. elegans* defense, we first used qRT-PCR to profile *zip-11* expression in the worms with deficiency in the major immune pathways respectively. Interestingly, we found that loss of PMK-1/p38 signal significantly suppressed *zip-11* mRNA level which did not differ between WT worms and DBL-1/TGF-β pathway (*dbl-1* and *sma-6*) or DAF-16/FOXO pathway (*daf-2* and *daf-16*) mutation worms (**Figure 3A**; **Figure S3A in Supplementary Material**). Moreover, while *P. aeruginosa* infection induced the expression and accumulation of ZIP-11 in intestinal nuclei, the suppression of PMK-1 by RNAi dramatically inhibited visible ZIP-11::GFP signal (**Figure 3B**). Additionally, the similar changes of ZIP-11 level was confirmed by western blot (**Figures 3C, D**). These results indicate that PMK-1 signal may regulate immune response in part by controlling the expression of ZIP-11.

We further used survival rate upon *P. aeruginosa* infection to investigate the genetic relationship between ZIP-11 and the major innate immune pathways. As expected, the attenuated resistance to *P. aeruginosa*-mediated killing of *zip-11(tm4554)* worms was suppressed by MAPK *pmk-1* RNAi (**Figure 3E**) but not *dbl-1* or *daf-16* RNAi (**Figures S3B, C in Supplementary Material**). The knockdown of MAPKK *sek-1* or MAPKKK *nsy-1* also eliminated the sensitive phenotype caused by *zip-11* mutation (**Figures S3D, E in Supplementary Material**). In addition, western blot analysis indicated that *zip-11(tm4554)* worms exhibited lower level of active PMK-1 than that of WT worms (**Figures 3F, G**). Furthermore, seven PMK-1 pathway dependent genes, *lys-1*, *F35E12.5*, *T24B8.5*, *clec-85*, *K08D8.5*, *F08G5.6* and *M02F4.7*, were down-regulated in *zip-11(tm4554)* worms relative to that in WT worms (**Figure 3H**). To confirm the *in vivo* role of ZIP-11 in the regulation of PMK-1 pathway, a transgenic transcriptional reporter strain of PMK-1 activity that expresses GFP under the control of *T24B8.5* promoter was introduced (22). We observed that the *T24B8.5p::GFP* signal in *zip-11* mutation or RNAi worms was lower than that in control worms when fed on *E. coli* or exposed to *P. aeruginosa* (**Figure 3I**; **Figures S3F, G in Supplementary Material**), suggesting ZIP-11 not only accelerates the inducible immunity, but also the constitutive immunity. The similar activation effect in another transcriptional reporter strain of PMK-1 activity, *F08G5.6p::GFP* (30), was observed as well (**Figure 3J**; **Figures S3H, I in Supplementary Material**). Thus, ZIP-11 promotes the expression of PMK-1-dependent genes by activating PMK-1.

Here, we reveal that PMK-1 signal can positively control ZIP-11 expression, indicating ZIP-11 lies downstream of PMK-1 pathway. On the other hand, ZIP-11 acts upstream of PMK-1 pathway in innate immune activation. These findings give us a notion that there may be a potential feedback regulatory loop between ZIP-11 and PMK-1 pathway.

### ZIP-11 Acts Together With CEBP-2 to Promote Immune Response Against *P. aeruginosa* PA14 Infection

**Figure 4:**
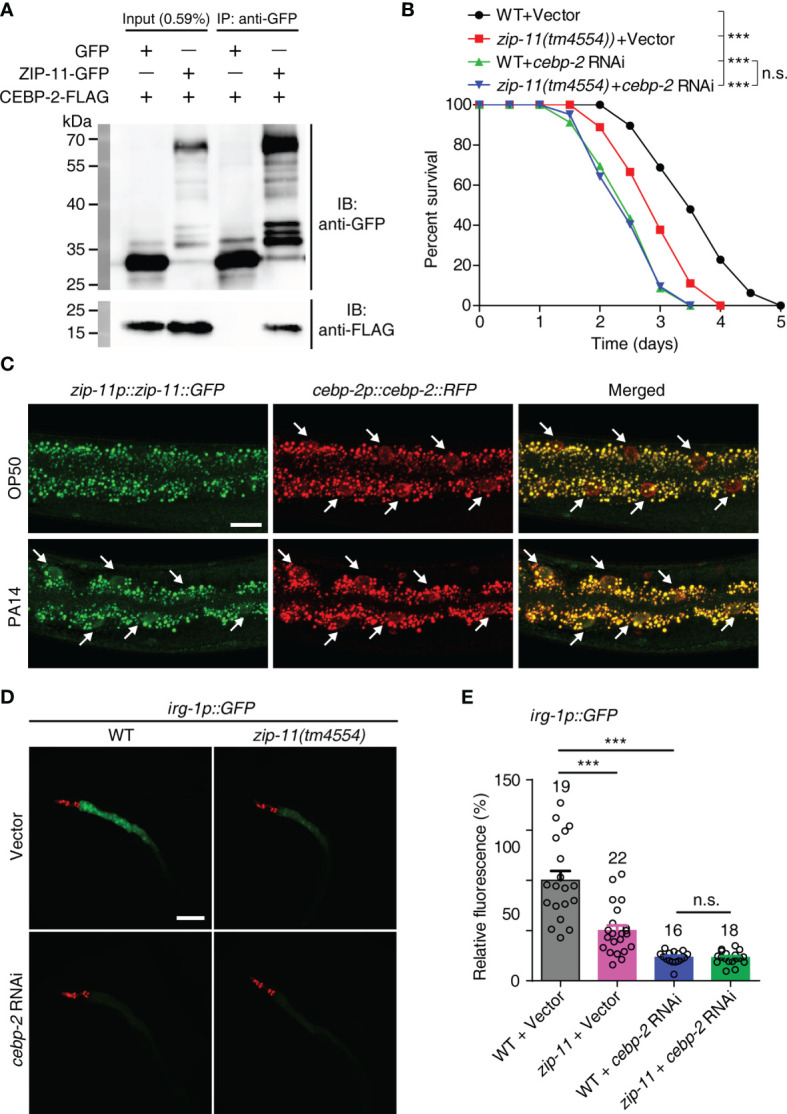
ZIP-11 interacts with CEBP-2 to regulate immune response against *P. aeruginosa* infection. **(A)** Immunoprecipitation of ZIP-11−GFP with anti-GFP antibodies was followed by western blot analysis with anti-Flag antibodies to detect the Flag-tagged CEBP-2. **(B)** WT worms and *zip-11(tm4554)* worms fed with vector control or *cebp-2* RNAi bacteria were exposed to *P. aeruginosa* PA14 and scored for survival. **(C)** Representative images of the *zip-11p::zip-11::GFP;cebp-2p::cebp-2::RFP* worms exposed to *E. coli* OP50 or *P. aeruginosa* PA14. ZIP-11::GFP or CEBP-2::RFP nuclear accumulation in the anterior portion of intestinal tissue (white arrows) are indicated. Scale bar: 20 μm. **(D)** Representative images of *irg-1p::GFP* and *zip-11(tm4554);irg-1p::GFP* worms fed with vector control and *cebp-2* RNAi bacteria were exposed to *P. aeruginosa* PA14. *myo-2p::mcherry* shows the red fluorescence of pharyngeal as a marker for the transgene. Scale bar: 200 μm. **(E)** Quantification of the GFP fluorescence intensity using the software program ImageJ. The number of animals analysed is indicated. Data are presented as mean ± SEM. Statistical significance was determined by log-rank test **(B)** or Student’s *t*-test **(E)**. ***p < 0.001 compared with respective controls unless specifically indicated; n.s., not significant. Please see **Table S4 in Supplementary Material** for detailed statistical analysis of killing assay data.

As bZIP proteins commonly act as dimers, we attempted to identify the interaction partner that interacts with ZIP-11 in *C. elegans* immune response. A previous study about bZIP transcription factor protein-protein interaction network *in vitro* identified *C. elegans* CEBP-2 (CCAAT/enhancer-binding protein 2) as the highest-affinity binding partner for ZIP-11 (31). Here, the coimmunoprecipitation (Co-IP) experiments was performed to investigate the binding relationship between ZIP-11 and CEBP-2. When we co-expressed tagged ZIP-11 and CEBP-2 in 293T cells, we were able to coimmunoprecipitate the two proteins as a complex (**Figure 4A**), suggesting that ZIP-11 and CEBP-2 can physically interact indeed. This interactive relationship was further revealed by GST pull-down assay (**Figure S4A in Supplementary Material**). In addition, the decrease in survival upon *P. aeruginosa* infection of *zip-11(tm4554)* worms was abolished by *cebp-2* RNAi (**Figure 4B**). Meanwhile, RNAi-mediated *zip-11* knockdown can’t further weaken the survival of *cebp-2(tm5421)* worms upon *P. aeruginosa* infection (**Figure S4B in Supplementary Material**), indicating that ZIP-11 and CEBP-2 may act in the same genetic pathway in immune regulation. However, *cebp-2*-defect worms exhibited decreased survival compared to *zip-11* mutants (**Figure 4B**), which may attribute to CEBP-2 can interact with various immune-related transcription factors, such as ZIP-2 (32), and plays a pivotal role in innate immune response. Although ZIP-11 and CEBP-2 are expressed in diverse tissues and *P. aeruginosa* infection can’t make change to CEBP-2, these two bZIP transcription factors exist noticeable co-localization in intestinal nucleus of worms upon *P. aeruginosa* infection (**Figure 4C**; **Figure S4C in Supplementary Material**).

We next examined whether ZIP-11 regulates the expression of target genes known to be induced by CEBP-2 upon *P. aeruginosa* infection. Considering the previous study have shown that an infection response gene-1 (*irg-1*) was robustly induced upon *P. aeruginosa* infection which process is controlled by ZIP-2 and CEBP-2 (27, 32), the reporter strain *irg-1p::GFP* was introduced to further investigate the relationship between ZIP-11 and CEBP-2 *in vivo*. Interestingly, upon *P. aeruginosa* infection, *zip-11* mutation significantly reduced the induction of *irg-1p::GFP* in vector control worms, but not in *cebp-2* RNAi-treated worms (**Figures 4D, E**), suggesting *irg-1* induction was controlled by ZIP-11 and CEBP-2. To a certain extent, this result also supports the equivalent survival between *cebp-2*- and *zip-11*-defect worms upon *P. aeruginosa* infection (**Figure 4B**; **Figure S4B in Supplementary Material**).

Our results showed that there is a connection between ZIP-11 and PMK-1/p38 pathway in innate immune response (**Figure 3**). However, the induction of *irg-1* upon *P. aeruginosa* infection was independent of the known innate immune pathways, including PMK-1/p38 pathway (27). To further investigate the relationship between CEBP-2 and PMK-1, *cebp-2* and *irg-1* mRNA levels were examined in WT and *pmk-1(km25)* worms by using qRT-PCR. We found that not only *irg-1* expression, but also *cebp-2* expression didn’t rely on PMK-1 signal (**Figure S5A in Supplementary Material**). Meanwhile, RNAi-mediated *cebp-2* knockdown further decreased the survival rate of *pmk-1(km25)* worms (**Figure S5B in Supplementary Material**), which could not alter the activation of PMK-1 (**Figures S5C, D in Supplementary Material**) or the GFP intensity of the two PMK-1 dependent reporters, *T24B8.5p::GFP* and *F08G5.6p::GFP* (**Figures S5E**–**H in Supplementary Material**), suggesting that CEBP-2-mediated immune regulation is independent of PMK-1/p38 pathway.

Taken together, these results indicate that ZIP-11 and CEBP-2 function together to regulate immune genes in the context of pathogenic bacteria infection, which process is independent PMK-1/p38 pathway.

### Human bZIP Transcription Factor ATF4 Functionally Substitutes for *C. elegans* ZIP-11

**Figure 5:**
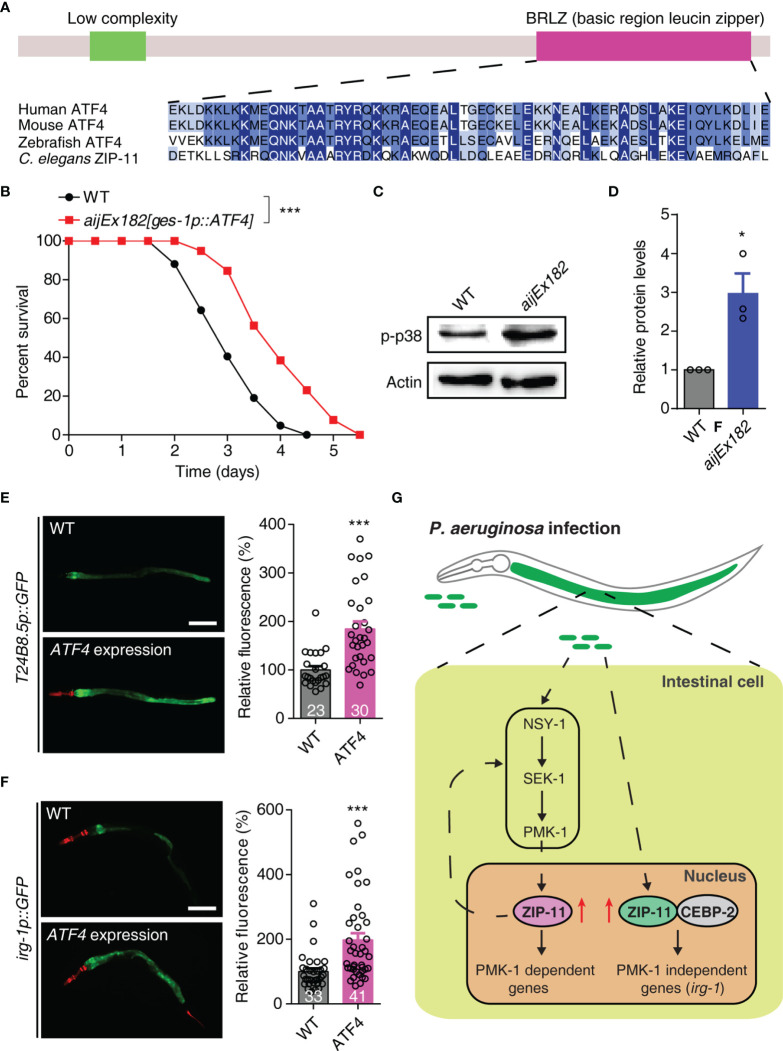
Human ATF4 can functionally substitute for *C. elegans* ZIP-11 in immune regulation. **(A)** Schematic of the *C. elegans* ZIP-11 protein showing the domain information predicted by SMART (http://smart.embl-heidelberg.de) (top) and the BRLZ domain multiple sequence alignment of ZIP-11 homologues from major metazoans. **(B)** WT and *aijEx182[ges-1p::ATF4]* worms were exposed to *P. aeruginosa* PA14 and scored for survival. **(C)** Western blot analysis of p38 phosphorylation levels in WT and *aijEx182[ges-1p::ATF4]* worms. **(D)** Quantification of the protein expression levels using the software program ImageJ. **(E)** Representative images and quantification of *T24B8.5p::GFP* in WT and ATF4-expressed worms. *myo-2p::mcherry* shows the red fluorescence of pharynx as a marker for the transgene. The number of animals analysed is indicated. Scale bar: 200 μm. **(F)** Representative images and quantification of *irg-1p::GFP* in WT and ATF4-expressed worms. *myo-2p::mcherry* (pharynx) and *lin-44p::mcherry* (tail) shows the red fluorescence as markers for the transgene. The number of animals analysed is indicated. Scale bar: 200 μm. **(G)** Proposed model of innate immune regulation by ZIP-11. Upon *P. aeruginosa* infection, the intestinal bZIP transcription factor ZIP-11 is induced, subsequently constitutes a feedback loop with PMK-1/p38 pathway and interacts with CEBP-2, further promote the expression of immune genes and enhance host defense. Data are presented as mean ± SEM. Statistical significance was determined by log-rank test **(B)** or Student’s *t*-test **(D–F)**. *p < 0.05, ***p < 0.001 compared with respective controls unless specifically indicated. Please see **Table S4 in Supplementary Material** for detailed statistical analysis of killing assay data.

Human activating transcription factor ATF4 (NP_001666.2), which acts as a master regulator and plays crucial role in the adaptation to stresses (33), is the top BLAST hit in the human protein database for *C. elegans* ZIP-11. ATF4 has a similar domain structure with ZIP-11 and most of the protein is composed of a bZIP domain. The BRLZ (basic region leucine zipper) domain of ZIP-11 shows a strong sequence similarity to that of the evolutionarily conserved basic leucine zipper transcriptional factor in eukaryotes (**Figure 5A**). We next expressed the human ATF4 as a transgene in WT worms using the intestine-specific promoter, and found that the transgene enhanced worm’s survival upon *P. aeruginosa* infection (**Figure 5B**). These results suggest that human ATF4 may play conserved function in innate immune regulation across species.

Given that ZIP-11 activates innate immune response through conserved PMK-1/p38 and CEBP-2 immune signals. We next investigated whether ATF4 expression induces the key components of these two pathways in *C. elegans*. As expected, western blot analysis showed that the ATF4-expressed worms had higher levels of active PMK-1 compared with control worms (**Figures 5C, D**). Furthermore, ATF4 expression significantly enhanced the GFP levels of PMK-1-dependent transgene worms, *T24B8.5p::GFP* (**Figure 5E**). In addition, human ATF4 also induced the expression of PMK-1-independent *irg-1* upon *P. aeruginosa* infection (**Figure 5F**). These results indicate that human ATF4 can activate PMK-1- and CEBP-2-dependent immune signals in *C. elegans*.

Combined, these data present convincing evidences supporting that human bZIP transcription factor ATF4 can functionally substitute for *C. elegans* ZIP-11 in innate immune regulation, raising the possibility that it may play a similar role in human.

## Discussion

When animals are exposed to microbial pathogens, the immune system will immediately activate a defense response through immune cells. Despite lacking professional immune cells, *C. elegans* can effectively protect itself from diverse invading pathogens, which largely relies on the innate immune response in epithelial cells. As a crucial immune organ, the epithelial cells can quickly recognize the pathogenic bacteria, inducing the antimicrobial peptides and secreted lysozymes to fight off the infection, which process is directly mediated by transcription factors. In the current study, we identified bZIP transcription factor ZIP-11 as a novel innate immune regulator (**Figure 5G**). ZIP-11 can constitute a feedback loop with PMK-1/p38 pathway. On the other hand, ZIP-11 acts together with another bZIP transcription factor, CEBP-2, to induce immune genes expression upon *P. aeruginosa* infection, which process is independent of PMK-1/p38 pathway. These findings reveal that ZIP-11 plays multiple functions in the complex immune regulatory network. Moreover, the overexpression of human ATF4 in worm’s intestine enhances the immune capacity of WT worms, promotes PMK-1 phosphorylation level, and induces the expression of PMK-1- or CEBP-2-dependent immune genes. Based on these findings, we proposed that the main function we identified for ZIP-11 in *C. elegans* may be evolutionarily conserved.

As one of the 12 members of the *zip* family in *C. elegans*, ZIP-11 has not been characterized yet. Despite the expression pattern of ZIP-11 was clarified by using the transcriptional worm *zip-11p::zip-11::SL2::GFP* that the GFP signal was visible in pharynx, hypodermis, and intestine (**Figure S2 in Supplementary Material**), *zip-11* mRNA was detected in the PVD neuron microarray profile (34). However, the tissue-specific RNAi and rescue assay indicate that only intestinal ZIP-11 is involved in the innate immune response (**Figure 2**). Since several transcriptome analyses showed ZIP-11 expression changed in response to multiple stress environments such as resveratrol treatment (35), rotenone treatment (36), or *S. marcescens* infection (37), ZIP-11 may modulate the global transcriptional regulation of stress response in *C. elegans*.

The p38 MAPK pathway is evolutionarily conserved in the control of cellular responses to stress and inflammation, and keeping it in balance seems critical to immune homeostasis (38). Previous studies have demonstrated that PMK-1/p38 pathway can receive various upstream signal to drive the “fight-or-flight” response and local immune response in *C. elegans* intestinal cells. In current study, we show that ZIP-11 acts as an immune activator by constituting a feedback loop with PMK-1/p38 in cell-autonomous manner. Though most immune response-related transcription factors are considered to directly modulate the expression levels of very downstream antimicrobial peptides and secreted lysozymes, which process is controlled by kinase signal. However, we didn’t clarify whether the PMK-1-dependent immune genes were directly controlled by ZIP-11. In addition, while SKN-1 and ATF-7 are two important transcription factors in the intestinal immune system of *C. elegans* that directly activated by NSY-1-SEK-1-PMK-1 MAPK signaling (20, 22), they may directly modulate ZIP-11 expression. Overall, the exact mechanism through which ZIP-11 activates PMK-1/p38 pathway and how PMK-1/p38 pathway promotes ZIP-11 expression remain unclear and require further study.

Although a series of molecules have been identified to be involved in regulating innate immune response in *C. elegans*, it is rarely investigated whether their functions are conserved in other organism. In human immune response, ATF4 activity is induced by the exposure of immune cells in multiple stressed environments including pathogen invasion, infection, inflammation, or tumor microenvironment (33). The regulation of immunity *via* ATF4 can be indirect by initiating inflammation that will secrete a series of cytokine directed immune responses. In contrast, ATF4 can also directly interfere with maturation, development, or polarization states of different immune cells such as macrophages, T cell, B cells, NK cells and dendritic cells contributing to progression of disease (39). Interestingly, ATF4 can act together with ATF6 to trans-activate the transcription factor C/EBP homologous protein (CHOP), which was found to regulate mediators of apoptosis such as B-cell lymphoma 2 (BCL2) and BCL2 interacting mediator of cell death (40, 41). Moreover, a previous study has shown that C/EBPγ, the human homolog of *C. elegans* CEBP-2, can dimerize with ATF4 to control redox homeostasis in normal and cancerous cells (42), suggesting the binding relationship between ZIP-11 and CEBP-2 is conserved. Overall, these excellent work further supports our notion that the immune regulation mechanisms of ZIP-11 we revealed in *C. elegans* might be applied in human. Future efforts are needed to explore the role of ATF4 in immune response of human, which may provide us a potential therapeutic target for the infectious diseases.

## Materials and Methods

### *C. elegans* and Bacterial Strains

*C. elegans* strains were provided by the Caenorhabditis Genetics Center (CGC),which were maintained with standard procedures unless otherwise specified (43). The Bristol strain N2 was used as the reference wild type. The following mutants and transgenic strains were used: *zip-11(tm4554)*, NU3 *dbl-1(nk3)*, LT186 *sma-6(wk7)*, CB1370 *daf-2(e1370)*, CF1038 *daf-16(mu86)*, KU25 *pmk-1(km25)*, KU4 *sek-1(km4)*, *cebp-2(tm5421)*, AU3 *nsy-1(ag3)*, OP761 *wgIs761[zip-11::TY1::EGFP::Flag + unc-119(+)]*, RW11749 *stlIs11749[cebp-2p::cebp-2::RFP]*, HTU388 *zip-11(tm4554);aijEx179[zip-11p::zip-11::SL2::GFP* + *myo-2p::mcherry]*, HTU392 *zip-11(tm4554);aijEx180[ges-1p::zip-11::SL2::GFP* + *myo-2p::mcherry]*, TU3311 *uIs60[unc-119p::YFP + unc-119p::sid-1]*, NR222 *rde-1(ne219);kzIs9*, WM118 *rde-1(ne300);neIs9*, DCL569 *rde-1(mkc36);mkcSi13*, VP303 *rde-1(ne219);kbIs7*, AU78 *agIs219[T24B8.5p::GFP]*, SAL148 *denEx26[F08G5.6p::GFP]*, HTU405 *zip-11(tm4554);agIs219[T24B8.5p::GFP]*, HTU406 *zip-11(tm4554);denEx26[F08G5.6p::GFP]*, AU133 *agIs17[irg-1p::GFP* + *myo-2p::mCherry]*, HTU431 *zip-11(tm4554);agIs17[irg-1p::GFP* + *myo-2p::mCherry]*, HTU432 *wgIs761[zip-11::TY1::EGFP::Flag + unc-119(+)];stlIs11749[cebp-2p::cebp-2::RFP]*.

### Plasmid Construction and Generation of Transgenic Lines

To construct plasmids pXT08 (pjet1.2_*zip-11p::zip-11::SL2::GFP::unc-54 3’-UTR*), pXT09 (pjet1.2_*ges-1p::zip-11::SL2::GFP::unc-54 3’-UTR*) or pXT10 (pjet1.2_*ges-1p::ATF4::SL2::GFP::unc-54 3’-UTR*), the 3.0 kb *zip-11* promoter or 2.5 kb *ges-1* promoter sequence, the *zip-11* or *ATF4* coding sequence, the *SL2::GFP* sequence, and *unc-54 3’-UTR* sequence were respectively subcloned into pjet1.2 between *Not*I and *Nco*I sites using isothermal assembly (44). These two plasmids were microinjected into *zip-11(tm4554)* worms at a concentration of 50 ng/μl along with *myo-2p::mcherry* at a concentration of 5 ng/μl to create the *zip-11(tm4554);aijEx179[zip-11p::zip-11::SL2::GFP]*, *zip-11(tm4554);aijEx180[ges-1p::zip-11::SL2::GFP]* and *zip-11(tm4554);aijEx183[ges-1p::ATF4::SL2::GFP]* transgenic lines. To construct plasmids pXT11 (pjet1.2_*ges-1p::ATF4::unc-54 3’-UTR*), the sequence with the deletion of the *SL2::GFP* was amplified by reverse PCR strategy and using pXT10 as a template, and then was self-fused using isothermal assembly. This plasmid was microinjected into WT worms at a concentration of 50 ng/μl along with *myo-2p::mcherry* at a concentration of 5 ng/μl to create the *aijEx182[zip-11p::ATF4]* transgenic line. For the construction of the plasmids pXT11 (pHT48_*zip-11::GFP*) or pXT12 (pHT48_*ATF4::3×Flag*), the *zip-11* or *ATF4* coding sequence, and the GFP or *3×Flag* sequence were respectively subcloned into pHT48 between *BamH*I and *Xho*I sites using isothermal assembly.

To construct plasmid pXT07 for *zip-11* RNAi, the 765 bp genomic sequence of *zip-11* (from the start codon to the end of the second exon) was subcloned into the control vector L4440 between *Not*I and *Hind*III using isothermal assembly.

### *C. elegans* Slow-Killing Assay

The slow-killing assay was performed as described previously (5) with minor modifications. The overnight culture of *P. aeruginosa* PA14 was seeded on modified NGM (0.35% instead of 0.25% peptone) in 35 mm-diameter plates. Plates were allowed to dry at room temperature and incubated at 37 °C for 24 h and at 25 °C for 8-24 h. Synchronized L4 worms were transferred to the *P. aeruginosa* PA14 slow killing plates and cultivated at 25 °C. Animals were scored at the indicated times for survival and transferred to fresh slow killing plates daily. Animals were considered dead if they failed to respond when touched. 5-Fluoro-2’-deoxyuridine (FUdR, 50 μg/ml) (Sigma, F0503) was included in the slow killing assay to prevent the growth of progeny and “bagging”. The worms died due to vulva burst or crawling off the plates were censored. Full-lawn *P. aeruginosa* PA14 plates were prepared for infection. All survival data were performed at least two replicates.

### Lifespan Assay on Heat-Killed *P. aeruginosa* PA14

*P. aeruginosa* PA14 was grown as described above. Bacteria were concentrated 1:5 and heat-inactivated at 65 °C. Synchronized L4 worms were transferred to the plates of modified NGM containing heat- inactivated *P. aeruginosa* PA14, 50 μg/ml FUdR and 50 μg/ml ampicillin (Sangon Biotech, A610028, China).

### *P. aeruginosa* PA14 CFU Assay

The worms were exposed to *P. aeruginosa* PA14 as described above. After infection for 24 h, worms were transferred to M9 solution containing 25 mM sodium azide (Amresco, 0639) to paralyze the worms and stop the pharyngeal pumping. Worms were washed three times with an antibiotic M9 solution containing 1 mg/ml ampicillin and 1 mg/ml gentamicin (Solarbio, G8170, China) followed by 30 min of incubation in the antibiotic solution to kill the bacteria present on the exterior of the worms. After transfer onto the new NGM plates containing 1 mg/ml ampicillin and 1 mg/ml gentamicin for another 30 min to further eliminate the external *P. aeruginosa* PA14, the worms were lysed with a motorized pestle. Lysates were serially diluted with M9 buffer and plated on Luria-Bertani plates containing 100 μg/ml rifampicin (Solarbio, R8011, China) to select for *P. aeruginosa* PA14. After overnight incubation at 37 °C, colonies of *P. aeruginosa* PA14 were counted to determine CFU per worm. Ten worms were examined per treatment, and at least three replicate assays were performed.

### Behavioral Analysis

Day 1 adult animals were used for the behavioral assays at room temperature. For the brood size assay, a single L4 worm was transferred to an individual plate. The worms were transferred to fresh plates every other day until laying ceased, and the offspring were subsequently scored for the brood size. For pumping rate assay, the pharyngeal pumping was determined directly under a digital automated microscope (Nikon, AZ100, Japan). For the defecation assay, the enteric muscle contractions (EMC) were scored for each defecation cycle using a dissecting microscope.

### RNA Isolation and Quantitative Real-Time PCR (qRT-PCR)

Synchronized L1 worms were placed onto NGM plates seeded with *E. coli* OP50 and grown at 20 °C until the L4 stage. Animals were collected and washed off the plates with M9 solution before being transferred to the modified NGM plates containing *E. coli* OP50 or *P. aeruginosa* PA14 for 24 h at 25 °C. Then, the animals were washed off the plates with M9 solution and frozen in TRIzol (Transgen, ER501-01, China). Total RNA was isolated using a TransZol Up Plus RNA kit (Transgen, ER501-01, China) and used for cDNA synthesis *via* an All-in- One first-strand cDNA synthesis SuperMix kit (Transgen, AT341-02, China). qRT-PCR was performed using SYBR PCR master mix (Transgen, AQ141-02, China) on a Bio-Rad CFX96 real-time PCR machine. Relative fold-changes for the transcripts were calculated using the comparative C_T_ (2^-ΔΔCT^) method and normalized to the *tba-1* gene, encoding a tubulin. All experiments were performed in triplicate.

Primer sequences used for qRT-PCR were *tba-1*: forward TCAACACTGCCATCGCCGCC and reverse TCCAAGCGAGACCAGGCTTCAG; *zip-2*: forward CGGATTGCCTGACGACTACA and reverse AATAACGGTCCGCTTTCGGT; *zip-10*: forward TCTGCTGCTCCTGGTAAA and reverse ATAAAGACGGCGATGACG; *zip-11*: forward CCGTAAACGCCAGCAAAACA and reverse TGAGCCGCTGATTACGATCC; *lys-1*: forward TTCGGATCTTTCAAGAAG and reverse TGGGATTCCAACAACGTA; *F35E12.5*: forward ACACAATCATTTGCGATGGA and reverse GGTAGTCATTGGAGCCGAAA; *T24B8.5*: forward TGCTTCAGAGTCGTGTGTCG and reverse ACGCAGACACCACAGGTTTT; *clec-85*: forward CCTGATGATAAGTATATT and reverse GGTTTTGGCTGTAGCACG; *K08D8.5*: forward TTACGATGGTGATTCCGT and reverse GCTTGTTGCCAGTTGAGA; *F08G5.6*: forward TGGACAACCCAGATATGCAA and reverse GTATGCGATGGAAATGGACA; *M02F4.7*: forward AAGCATGGGAGGAACACTGG and reverse CTGTCGCGGTAGAAACCCAA; *cebp-2*: forward AAGCTGTGAACAGAACCCGA and reverse CTGTAGCTGCTCGACCTTTCT; *irg-1*: forward GACATGGACATCGCAGAGCA and reverse TGCGCGGTACATTACATCCT.

### RNA Sequencing

Total RNA was obtained as described above. 1 μg total RNA from each sample was used as input material for the RNA sample preparations. Sequencing libraries were generated using NEBNext^®^ Ultra™ RNA Library Prep Kit for Illumina^®^ (NEB, USA) following manufacturer’s recommendations and index codes were added to attribute sequences to each sample. After cluster generation on a cBot Cluster Generation System using TruSeq PE Cluster Kit v3-cBot-HS (Illumina), the library preparations were sequenced on an Illumina Hiseq platform and 125 bp/150 bp paired-end reads were generated.

The clean reads were mapped to the *C. elegans* genome sequence using Hisat2 v2.0.4. HTSeq v0.9.1 was used to count the reads numbers mapped to each gene. And then FPKM of each gene was calculated based on the length of the gene and reads count mapped to this gene. Differential expression analysis of two groups (three biological replicates per condition) was performed using the DESeq R package (1.18.0). The resulting p-values were adjusted using the Benjamini and Hochberg’s approach for controlling the false discovery rate. Genes with an adjusted p-value < 0.05 found by DESeq were assigned as differentially expressed.

### RNA Interference

RNAi was performed by feeding worms with *E. coli* strain HT115 (DE3) expressing double-stranded RNA homologous of target genes as described (45). HT115 (DE3) was grown overnight in LB broth containing 100 μg/ml ampicillin at 37 °C and plated over the NGM plates containing 100 μg/ml ampicillin and 2mM isopropyl 1-thio-β-D-galactopyranoside (IPTG) (Sangon Biotech, A600168). RNAi-expressing plates were allowed to grow for approximately 24 h at room temperature. The L2-L3 larval worms of the corresponding strain were placed on the RNAi plates for 2 days at 20 °C. The gravid adults were then transferred onto the fresh RNAi-expressing bacterial lawns and allowed to lay eggs for 2 h to obtain the second generation of RNAi population. The eggs were allowed to develop at 20 °C to reach the L4 stage for subsequent experimental use. In all experiments, *uaf-1* RNAi was included as a positive control to account for RNAi efficiency.

### Immunoblotting, Coimmunoprecipitation and GST Pull-Down Assay

The worm and cell lysate samples were prepared with the lysis buffer in the presence of protease inhibitors (Sigma-Aldrich, 10253286001, L2884-1MG, 10236624001, USA) or protease and phosphatase inhibitors (Sigma-Aldrich, 04906845001, USA). Proteins were detected using the following antibodies: a rabbit anti-GFP polyclonal antibody (Invitrogen, A-11122, USA), a rabbit anti-phospho-p38 MAPK monoclonal antibody (Cell Signaling Technology, 4511, USA), and a mouse anti-β-actin monoclonal antibody (Transgen, HC201-01, China).

Cell transfection and coimmunoprecipitation (Co-IP) were performed as previously described (46). Briefly, 293T cells (CRL-11268, ATCC) were maintained in DMEM medium (Gibco, C11995500BT, USA) with 10% inactive bovine fetal serum (Gibco, 10270106, USA). The cells were transiently transfected with the plasmids expressing CEBP-2-Flag, ZIP-11-GFP, or GFP by using Lipofectamine 3000 transfection reagent (Invitrogen, L3000015, USA). The cells were harvested at around 48 hours post-transfection and washed two times to remove the cell debris (800 g, 3 min). The cells were lysed with ice pre-cooled immunoprecipitation buffer (IPB, 50 mM HEPES, pH 7.7, 100 mM NaCl, 50 mM KCl, 2 mM MgCl_2_, 2 mM CaCl_2_, 1 mM EDTA, 1 μg/ml pepstatin, 1 μg/ml leupeptin, 2 μg ml-1 aprotinin, 1 mM PMSF) containing 1% Triton X-100 on ice for 60 min, then centrifuged at 20000 g for 10 min at 4°C. Total protein content was estimated by PierceTM BCA Protein Assay Kit (Thermo-Scientific, 23227, USA) following the manufacturer’s protocol. The protein was diluted with IPB (with 0.05% Triton X-100) to a final concentration of 1 ug/ul. The prewashed 20 μl of anti-GFP-Trap-A beads (Chromotek, gta-20, Germany) was added and incubated overnight at 4°C with gentle rotation. The anti-GFP-Trap-A beads were collected by centrifugation at 1000 g for 3 min at 4°C. The beads were washed six times with IPB containing 0.05% (w/v) Triton X-100. After last time wash, the beads were resuspended with 60 μL IPB buffer, the immunoprecipitated proteins were boiled in 3 × Sample buffer with beta-mercaptoethanol for 10 min at 98°C, and analyzed by western blotting. For the western blotting, the primary anti-GFP Rabbit polyclonal antibody (Invitrogen, A-11122, USA) at a 1:3000 dilution or anti-Flag mouse monoclonal antibody (MBL, M185-3L, USA) at a 1:12000 dilution was used, and horseradish peroxidase (HRP)-conjugated goat anti-rabbit (Transgen, HS101-01, China) or anti-mouse (Transgen, HS201-01,China) was used as secondary antibody at a 1:2000 or 1:5000 dilution. The images of blotting membranes were captured by an imaging system (MicroChemi 4.2, DNR Bio Imaging Systems). The coimmunoprecipitation experiment was repeated at least three times independently.

To confirm the interaction of CEBP-2 and ZIP-11, CEBP-2 was expressed as GST-fusion proteins in pGEX-4T-1, and the HEK293T cells were transfected with ZIP-11-GFP plasmid. The cells and bacteria were collected and extracted with working buffer (WB, 50 mM HEPES, pH7.4, 100 mM NaCl, 50 mM KCl, 2 mM CaCl_2_, 2 mM MgCl_2_, 1 mM EDTA, 1 μg/mL pepstatin, 1 μg/mL leupeptin, 2 μg/mL aprotinin, 1mM PMSF, 1 tablet of protease inhibitor cocktail per 10 mL buffer) containing 0.5% Triton X-100. Equal amounts of GST-fused CEBP-2 or GST protein was added to the cell lysate. Then the pre-washed 25 μl of GST-beads was added to the system, and incubated overnight at 4°C with gentle rotation. The GST-beads were collected by centrifugation at 700 g for 3 min at 4°C. The beads were washed six times with ice cold WB buffer containing 0.1% Triton X-100. After last time wash, the beads were resuspended by 70 μL WB buffer, the pull-downed proteins were boiled in 3 × Sample buffer with beta-mercaptoethanol for 10 min at 98°C, and analyzed by western blotting. For the western blotting, the primary anti-GFP Rabbit polyclonal antibody (Invitrogen, A-11122, USA) at a 1:3,000 dilution or anti-GST mouse monoclonal antibody (Transgen, HT601-01, China) at a 1:1,000 dilution was used, and horseradish peroxidase (HRP)-conjugated goat anti-rabbit (Transgen, HS101-01, China) or anti-mouse (Transgen, HS201-01,China) was used as secondary antibody at a 1:2000 or 1:5000 dilution. The images of blotting membranes were captured by the imaging system. The pull down experiment was repeated at least three times independently.

### Fluorescent Microscopy

To observe *zip-11p::zip-11::GFP*, *zip-11p::GFP* or *cebp-2p::cebp-2::RFP* expression, L4 worms were exposed to *E. coli* OP50 or *P. aeruginosa* PA14 for 8 to 24 h and paralyzed with 1mM levamisole. The animals were mounted on the agar pad on a slide. An ultra-high resolution spectral confocal microscope (OLYMPUS, FV1000, Japan) was used to capture images. To observe *T24B8.5p::GFP*, *F08G5.6p::GFP* or *irg-1p::GFP* expression, he digital automated microscope (Nikon, AZ100, Japan) was used to capture images. Exposure was constant in each experiment.

### Statistical Analysis

Data were presented as mean ± SEM. Survival curves were created using GraphPad Prism 8 (GraphPad, CA). The log-rank (Kaplan-Meier) test was used to analyze the survival data. For fluorescence quantification, the worms in the images were outlined and the signal intensity was calculated by the ImageJ software (NIH, USA). Other data were analyzed by using an unpaired Student’s t-test. The difference was considered significant from control for p-value < 0.05. All experiments were performed at least three times independently.

## Data Availability Statement

The datasets presented in this study can be found in online repositories. The names of the repository/repositories and accession number(s) can be found in the article/**Supplementary Material**.

## Author Contributions

ZZ, JL, and HT designed the experiments. ZZ, JL, YA, MA, SY, XZ, YX, and CC performed experiments. ZZ, JL, YA, and HT analyzed the data. ZZ, JL, and HT wrote the manuscript. All authors contributed to the article and approved the submitted version.

## Funding

This work was supported by National Key Research and Development Program of China (2021YFA090164 to HT), the National Natural Science Foundation of China (31800857 to CC), Key Project of Research and Development Plan of Hunan Province (2020SK2092 to HT), Provincial Natural Science Foundation of Hunan Province (2021JJ30098 to HT), the Foundation of Shenzhen Science and Technology Innovation Committee (JCYJ20210324121000001).

## Conflict of Interest

The authors declare that the research was conducted in the absence of any commercial or financial relationships that could be construed as a potential conflict of interest.

## Publisher’s Note

All claims expressed in this article are solely those of the authors and do not necessarily represent those of their affiliated organizations, or those of the publisher, the editors and the reviewers. Any product that may be evaluated in this article, or claim that may be made by its manufacturer, is not guaranteed or endorsed by the publisher.
