# Supplementary material for: The bZIP Transcription Factor ZIP-11 Is Required for the Innate Immune Regulation in Caenorhabditis elegans
Source: Front Immunol. 2021 Nov 5;12:744454. doi: 10.3389/fimmu.2021.744454 (PMC8602821; doi:10.3389/fimmu.2021.744454)
Supplement: Supplementary file 1 [file DataSheet_1.pdf]

Supplementary Material

Supplementary Tables and Figures

Supplementary Table 1. The up-regulated genes upon *P. aeruginosa* PA14 infection.
Supplementary Table 2. The down-regulated genes upon *P. aeruginosa* PA14 infection.
Supplementary Table 3. The expression level of *zip* class genes upon *P. aeruginosa* PA14 infection.
Supplementary Table 4. Statistics for survival analysis.
Supplementary Figure 1-5.

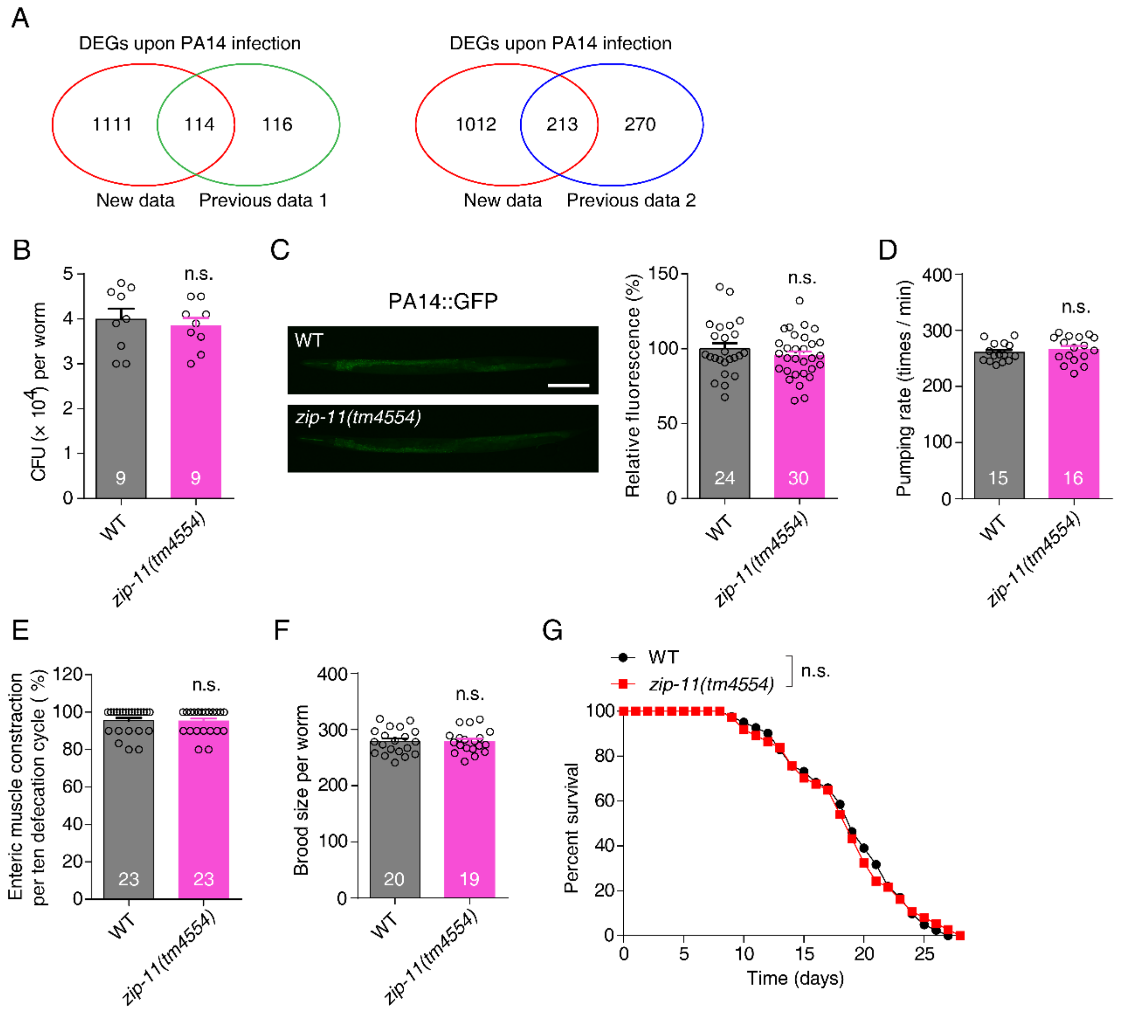

Supplementary Figure 1. The absence of ZIP-11 doesn't affect worm's behaviors associated with innate immune ability. **(A)** Venn diagrams indicating numbers of differentially expressed genes upon *P. aeruginosa* PA14 infection between this study and previous published data. **(B)** WT and *zip-11(tm4554)* worms were exposed to *P. aeruginosa* PA14 for 24 h and the colony-forming units (CFU) were counted. **(C)** Representative images and quantification of WT and *zip-11(tm4554)* worms fed with PA14::GFP bacteria for 24 h. **(D)** The pumping rates of WT and *zip-11(tm4554)* worms over 1 min were recorded before infection with *P. aeruginosa* PA14. **(E)** The enteric muscle contraction (EMC) cycle of normal cultured WT and *zip-11(tm4554)* worms were determined. **(F)** The brood size of normal cultured WT and *zip-11(tm4554)* worms were determined. **(G)** WT and *zip-11(tm4554)* worms were exposed to heat-killed *P. aeruginosa* PA14 and scored for survival. The number of animals analysed is indicated. Data are presented as mean ± SEM. Statistical significance was determined by log-rank test **(G)** or Student's *t*-test **(B, C, D, E and F)**. Please see Table S4 in Supplementary Material for detailed statistical analysis of killing assay data.

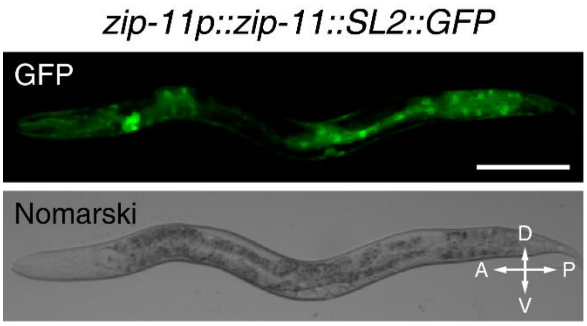

Supplementary Figure 2. The expression pattern of *zip-11*. Representative photomicrographs of *zip-11p::GFP* worms raised on *E. coli* shows *zip-11* is expressed in intestine, pharynx, and hypodermis. Scale bar: 200 µm.

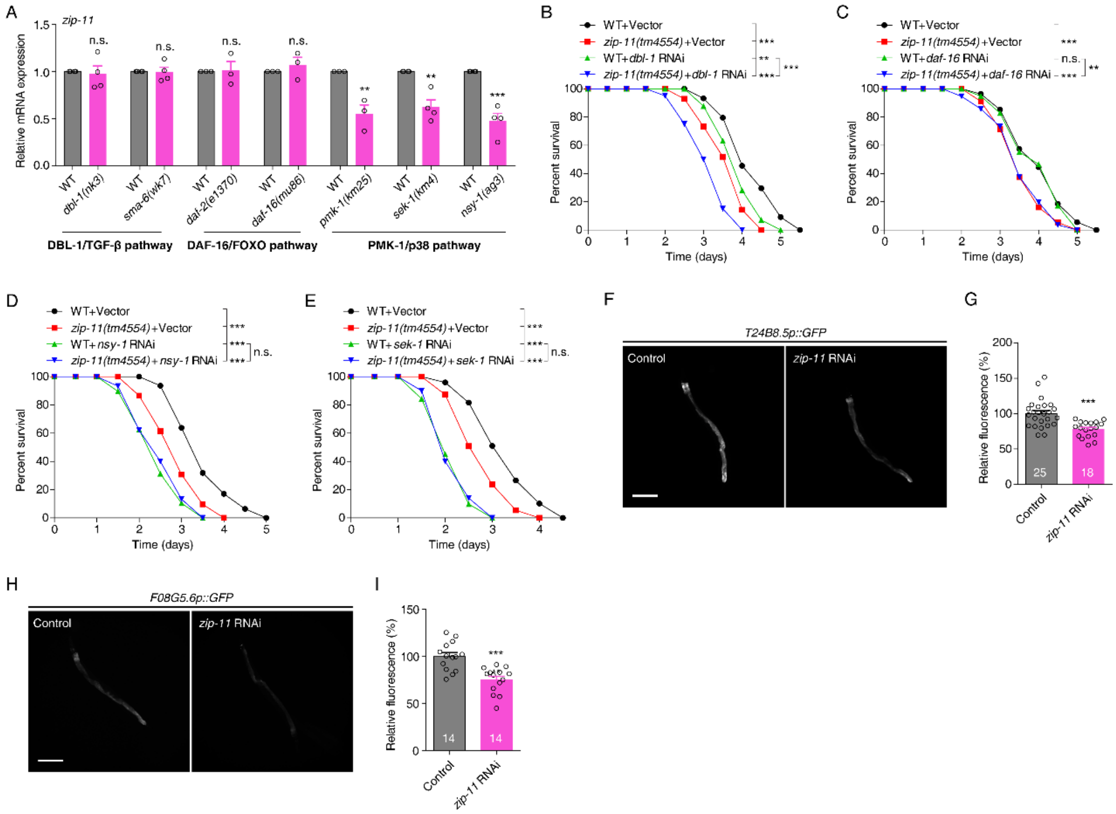

Supplementary Figure 3. ZIP-11 activates innate immunity via PMK-1/p38 pathway. **(A)** qRT-PCR analysis of *zip-11* expression levels in DBL-1/TGF-β pathway (*dbl-1* and *sma-6*), DAF-16/FOXO pathway (*daf-2* and *daf-16*), and PMK-1/p38 (*pmk-1*, *sek-1*, and *nsy-1*) mutation worms. **(B-E)** WT worms and *zip-11(tm4554)* worms fed with vector control, *dbl-1* RNAi **(B)**, *daf-16* RNAi **(C)**, *nsy-1* RNAi **(D)**, or *sek-1* RNAi **(E)** bacteria were exposed to *P. aeruginosa* PA14 and scored for survival. **(F-I)** Representative images and quantification of *T24B8.5p::GFP* **(F, G)** and *F08G5.6p::GFP* **(H, I)** in vector control or *zip-11* RNAi bacteria fed worms. Scale bar: 200 µm. The number of animals analysed is indicated. Data are presented as mean ± SEM. Statistical significance was determined by log-rank test **(B-E)** or Student's *t*-test **(A, G and I)**. $^{**}p < 0.01$, $^{***}p < 0.001$ compared with respective controls unless specifically indicated. Please see Table S4 in Supplementary Material for detailed statistical analysis of killing assay data.

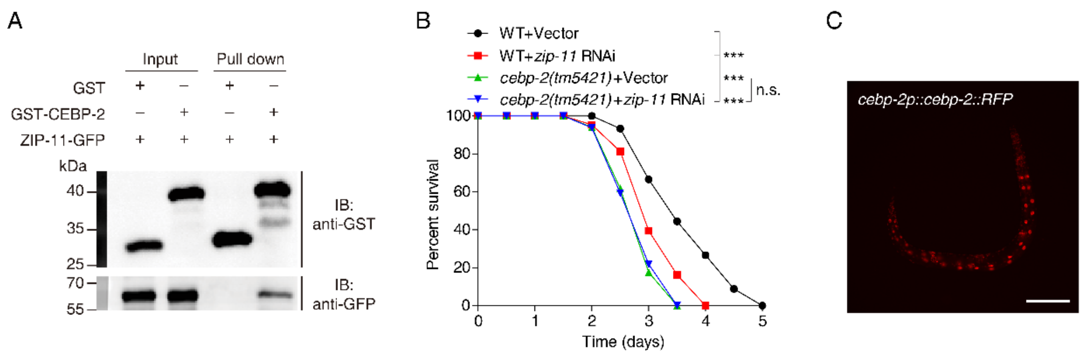

Supplementary Figure 4. ZIP-11 interacts with CEBP-2 to regulate immune response against *P. aeruginosa* infection. **(A)** GST pull-down of GST-CEBP-2 with anti-GST antibodies was followed by western blot analysis with anti-GFP antibodies to detect the GFP-tagged ZIP-11. **(B)** WT and *cebp-2(tm5421)* worms fed with vector control or *zip-11* RNAi bacteria were exposed to *P. aeruginosa* PA14 and scored for survival. **(C)** Representative photomicrograph of *cebp-2p::cebp-2::RFP* worms raised on *E. coli*. Scale bar: 200 µm. Statistical significance was determined by log-rank test **(B)**. Please see Table S4 in Supplementary Material for detailed statistical analysis of killing assay data.

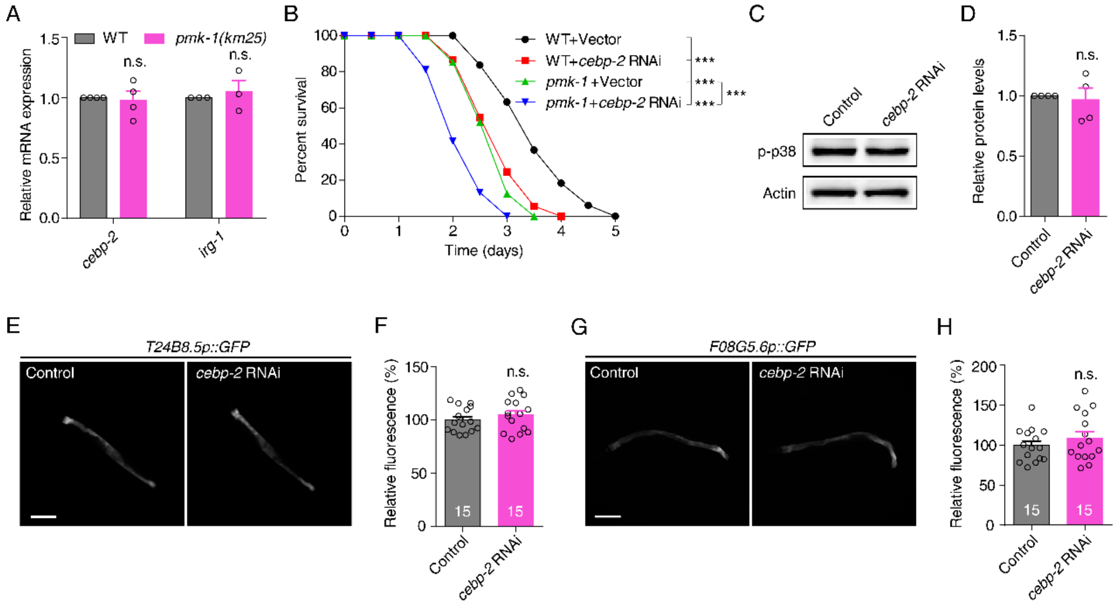

Supplementary Figure 5. CEBP-2-mediated immune regulation is independent of PMK-1/p38 pathway. **(A)** qRT-PCR analysis of *cebp-2* and *irg-1* expression levels in WT and *pmk-1(km25)* worms. **(B)** WT worms and *pmk-1(km25)* worms fed with vector control or *cebp-2* RNAi bacteria were exposed to *P. aeruginosa* PA14 and scored for survival. **(C)** Western blot analysis of p38 phosphorylation levels in WT worms fed with vector control or *cebp-2* RNAi bacteria. **(D)** Quantification of the protein expression levels using the software program ImageJ. **(E-H)** Representative images and quantification of *T24B8.5p::GFP* **(E, F)** and *F08G5.6p::GFP* **(G, H)** in vector control or *cebp-2* RNAi bacteria fed worms. Scale bar: 200 µm. The number of animals analysed is indicated. Data are presented as mean ± SEM. Statistical significance was determined by log-rank test **(B)** or Student's *t*-test **(A, D, F and H)**. Please see Table S4 in Supplementary Material for detailed statistical analysis of killing assay data.
